# Prevalence of Use, Impact on Oral Health, and Knowledge Regarding Tobacco Smoking: Findings from a Cross-Sectional Survey in Military Marines

**DOI:** 10.3390/ijerph23050655

**Published:** 2026-05-14

**Authors:** Siti Sopiatin, Yun Mukmin Akbar, Irvan Nur Wachid, Sharifa Ezat Wan Puteh, Neily Zakiyah, Amaliya Amaliya, Achmad Syawqie

**Affiliations:** 1 Biotechnology Doctoral Study Program, Graduate School, Universitas Padjadjaran, Bandung 40132, West Java, Indonesia; siti.sopiatin@unpad.ac.id; 2 Department of Periodontics, Faculty of Dentistry, Universitas Padjadjaran, Bandung 40132, West Java, Indonesia; 3 Department Dental and Oral Health, Dr. Mintohardjo Naval Hospital, Jakarta 10210, Indonesia; yunnakbar@yahoo.com; 4 Marine Health Battalion Unit 1/Marine Force 1, Jakarta 12560, Indonesia; irvan24003@mail.unpad.ac.id; 5 Department of Community Health, Faculty of Medicine, Universiti Kebangsaan Malaysia, Kuala Lumpur 43600, Malaysia; sh_ezat@hctm.ukm.edu.my; 6 Department of Pharmacology and Clinical Pharmacy, Faculty of Pharmacy, Universitas Padjadjaran, Bandung 45363, West Java, Indonesia; neily.zakiyah@unpad.ac.id; 7 Department of Oral Biology, Faculty of Dentistry, Universitas Padjadjaran, Bandung 40132, West Java, Indonesia; achmad.syawqie@unpad.ac.id

**Keywords:** smoking, oral health, knowledge, marines, military, survey

## Abstract

**Highlights:**

**Public health relevance—How does this work relate to a public health issue?**

- High smoking prevalence in the military is a significant public health issue, with rates often higher than in the general population.
- The length of military service and low education level influence smoking behavior.

**Public health significance—Why is this work of significance to public health?**

- Reduced operational readiness and fitness.
- Costs the military billions in lost productivity and increased healthcare expenses.

**Public health implications—What are the key implications or messages for practitioners, policy makers and/or researchers in public health?**

- Need for tobacco cessation and harm reduction policy in military service.
- Implement comprehensive smoke-free policies in all military buildings, housing, and vehicles.

**Abstract:**

Background: Despite well-documented adverse impact on both systemic and oral health, tobacco smoking remains a persistent issue in military populations. It contributes to the global burden of tobacco use and is often perceived as a means of coping with stress in military settings. Purpose: This study aimed to assess the prevalence of tobacco use among military marines, its impact on oral health, and their level of knowledge regarding smoking, as well as to identify variables associated with their smoking habits. Thus, it provides a basis for implementing appropriate tobacco cessation and harm reduction strategies, particularly within the military. This study demonstrated a high prevalence of tobacco use among military marines, despite generally high levels of knowledge regarding tobacco smoking. A knowledge gap was still evident in relation to smoking behavior. The most frequently reported oral health impacts among smokers were tooth staining, halitosis, and taste impairment. Duration of military service and level of knowledge were significantly associated with smoking behavior. Materials and Methods: A validated and reliable online survey was administered to collect socio-demographic data, including age, education level, and length of military service. The survey also assessed smoking status, smoking behavior, its impact on oral health, and participants’ knowledge of smoking-related risks. Data were analyzed descriptively, and associated factors were examined using multivariate analysis. Results: A total of 475 military marines participated in the study. Of these, 44.8% were current smokers, 25.7% were former smokers, and 29.5% had never smoked. Overall, 71% of participants demonstrated good knowledge of smoking-related risks. The most commonly reported oral health impacts were halitosis, tooth staining, and impaired taste. Smoking status did not differ significantly by age (*p* = 0.095) or education level (*p* = 0.610), but differed significantly by length of military service (*p* < 0.05) and level of knowledge (*p* < 0.05). Multivariate analysis using multinomial logistic regression indicated that length of military service was a significant predictor of smoking behavior (*p* = 0.005; 95% CI: 0.282–0.800), with 1–5 years of service emerging as the most influential category. Based on the odds ratio, individuals with 11–15 years of service had a 1.8-fold higher likelihood of smoking. Conclusions: Despite a generally good level of knowledge regarding the health risks of smoking, the prevalence of tobacco use remains high among military marines. The most commonly reported oral health impacts were tooth staining, halitosis, and impaired taste. Length of military service and level of knowledge regarding smoking were identified as significant factors associated with smoking status.

## 1. Introduction

Tobacco smoking remains a significant global health issue, with smoking prevalence varying widely across countries. Global Adult Tobacco Survey reported that from 28 countries, the median prevalence of tobacco smoking was 22.5% among persons aged ≥15 years [1]. Although the negative health impacts of smoking have long been well established, knowledge regarding its health risks remains underestimated, particularly among current smokers. While general awareness that smoking is harmful is high, a significant gap persists in understanding specific and relative risks. The habit of tobacco smoking is a major behavioral risk factor for numerous health outcomes, affecting both systemic and oral health. These include coronary heart disease, stroke, and multiple types of cancer, including lung cancer. In the oral cavity, smokers experience an 80% increased risk of periodontitis, a 36.6% higher prevalence of caries, and an elevated risk of oral cancer [2].

The use of tobacco is often closely associated with military service, as it is commonly perceived as a stress reliever; thus, it contributes to smoking prevalence globally. Military personnel experience more stressful conditions than the general population. A higher prevalence of smokers in military settings can be expected.

In the US military, tobacco use was 15.0% higher than among civilians, with an overall rate of 28.26% in 2022. In a Russian military study, 48.7% of military personnel were identified as active smokers, with the highest rates reaching 56.6% [3,4]. The prevalence of smoking among national military service recruits in the UAE was 41.6% [5]. Smoking rates in Uganda were substantially higher in the military (34.8%) compared to the general public (5.3%) [6]. In Germany, military smoking rates were slightly above those of the general population, while in Israel, smoking prevalence increased by 39.4% during military service [7].

Previous studies have indicated a high prevalence of tobacco use in military populations, particularly in Naval services at 24.1% [8]. In the U.S. Navy, 24.5% were reported as current smokers, whereas in the Taiwan Navy, the prevalence was 32.8% [9]. In the Chinese Navy, smoking rates were higher than in civilian and other military populations, reaching 56.14%. There are significant gaps between the knowledge regarding smoking among military personnel and the persistently high prevalence rates [10]. The vast majority of military personnel underestimate their personal health risks related to tobacco smoking [11]. The implementation of harm reduction strategies, including approaches that increase knowledge about the health risks posed by tobacco use, may help reduce tobacco use and tobacco-related diseases, including heart disease, stroke, lung cancer, and oral conditions, particularly among active military personnel [11].

This present study aimed to assess the prevalence of tobacco use among military marines working in a highly stressful environment, its impact on oral health, and their level of knowledge regarding smoking, as well as to identify variables associated with their smoking habits. Thus, it provides a basis for implementing appropriate tobacco cessation and harm reduction strategies, particularly within the military.

## 2. Materials and Methods

### 2.1. Participants and Procedures

This survey was conducted using an online questionnaire. Convenience sampling was applied among military marines who were able to access the survey online and were willing and able to complete the questionnaire. The study protocol was approved by the Ethics Committee of the Faculty of Medicine, Universitas Padjadjaran, Bandung, Indonesia (No. 1065/UN6.KEP/EC/2023) with a research permit from Naval Dental Hospital RE Martadinata, Jakarta, Indonesia (B/627/VII/2023).

Subjects were required to meet the inclusion criteria, namely being male or female, at least 18 years of age, active marine unit members, capable of completing the questionnaire voluntarily, and free from coercion by any individual. The exclusion criteria included military marines who did not agree to participate in the research procedures. Before completing the questionnaire, informed consent was obtained from all participants, indicating their agreement to participate in the study. Access to the survey was closed after the predetermined target number of respondents had been reached.

The questionnaire was developed based on a previous study [10] and was structured into sections covering smoking impact on oral health and knowledge related to smoking habits. Prior to the commencement of the study, the questionnaire underwent validity and reliability testing, was piloted with 30 respondents, and was designed to be completed within approximately 5 to 10 min. The validity test was performed with Pearson Product-Moment, and the reliability test with Kuder–Richardson (KR20). The results of the validity test for this research questionnaire compared the item-total correlations with the total correlations obtained. For the knowledge variable, all items regarding cigarettes scored above 0.300; therefore, these items were considered significant, had good validity, and could be used for further analysis. Based on the reliability test calculations, the knowledge variable yielded a correlation coefficient above 0.8, specifically 0.934. This indicates that the instrument was highly reliable, and thus suitable for further analysis.

### 2.2. Subject’s Characteristic

Smoking status in the present report was categorized into several groups: current smokers, former smokers, and never smokers. Participants’ sociodemographic characteristics were collected, including age, gender, educational level, and duration of military service.

### 2.3. Current Smoker Behavior and Cessation Attempts

This section was administered only to current smokers. It included seven questions regarding smoking behaviors, concerning the type of cigarette, duration, frequency, total smoking time, number of cigarettes consumed, and purpose of smoking. Current smoker cessation attempts and history were explored through five questions, including whether they had ever tried to stop smoking, whether they had received counseling for smoking cessation, whether they were confident in their ability to cease smoking within six months, whether they had perceived any negative effects, and their health expectations regarding their cessation attempts.

### 2.4. Former Smoker Behavior

This section was collected only from former smokers, and consisted of three questions (1) how long it had been since they ceased smoking, (2) how long they had been active smokers, and (3) the type of cigarettes consumed.

### 2.5. Assessment of Oral Health

Impact on oral health due to smoking was assessed using seven questions covering halitosis, tooth staining, impaired taste, delayed wound healing, and gingival impacts, including soreness, bleeding, and swelling.

### 2.6. Knowledge Regarding Smoking and Combustion-Free Nicotine Delivery System (C-F NDS)

Participants’ knowledge about smoking was explored through 14 questions, including the impact of smoking on oral cavity conditions and on systemic diseases that may result from smoking. Respondents’ knowledge of C-F NDS consisted of 3 questions: whether they had received information about C-F NDS, whether they recognized these products, and the variety of these products.

### 2.7. Statistical Analysis

Data were analyzed using descriptive statistics, reported as frequency distributions. Comparative tests between sociodemographic characteristics and knowledge level regarding smoking were further conducted to examine differences in factors related to smoking status. Factors with significant *p*-values (*p* < 0.05) were subsequently examined to determine their associations with the smoking habit of current smokers. The significant variables associated with current smoking were then analyzed using multivariate multinomial logistic regression.

The independent variable was smoking status, categorized as follows: current smokers (those who had smoked ≥100 cigarettes in their lifetime and still smoked up to the present), former smokers (those who had smoked ≥100 cigarettes in their lifetime but no longer smoked), and non-smokers (those who had never smoked ≥100 cigarettes in their lifetime). The dependent variables were age, education level, military service, and knowledge level regarding smoking. SPSS software version 23.0 was employed, and the level of significance was set at *p* < 0.05.

## 3. Results

### 3.1. General Characteristics

A total of 475 participants completed the survey. The socio-demographic characteristics of the participants are presented in Table 1, including prevalences of 44.8%, 25.7%, and 29.5% for current smokers, former smokers, and never smokers, respectively.

### 3.2. Smoking Behavior

The smoking behavior of participants in the present study is summarized in Table 2. The majority of both current smokers and former smokers used conventional/combustible cigarettes, while only a small proportion used vapes/e-cigarettes. Most current smokers had been smoking for more than 10 years, smoked 5–10 times a day, and spent 1–2 h with 5–10 cigarettes. Most smokers reported smoking without any specific purpose, while others indicated stress relief and improved focus and concentration during work as their primary reasons.

### 3.3. Cessation Attempts and History of Current Smokers

Almost all current smokers in this survey (Table 3) had attempted to quit smoking. Most had received advice to quit smoking and believed that they could quit within the next six months. More than half of the smokers reported experiencing adverse health effects related to smoking and agreed with statements regarding the expected health benefits of smoking cessation.

### 3.4. Impact on Oral Health Regarding Smoking

As presented in Table 4, stained teeth and halitosis were the most commonly reported impacts on oral health regarding smoking among current and former smokers. Approximately half of the respondents experienced impaired taste, while about one-third reported delayed wound healing, including gingival soreness, gingival bleeding, and gingival swelling.

### 3.5. Knowledge Regarding Smoking

Participants’ knowledge regarding smoking was assessed using a questionnaire (Table 5) with dichotomous responses (Yes or No). Correct answers were assigned 1 point, while incorrect answers were assigned 0 points. The total score was calculated by summing all items, ranging from 0 to 16. Scores of 0–5 indicated poor knowledge, 6–11 indicated fair knowledge, and 12–16 indicated good knowledge. Table 5 reveals that most of the respondents in this study demonstrated good knowledge regarding smoking.

### 3.6. Knowledge Regarding Non-Combustible Tobacco Products

Table 5 shows that six out of ten participants had heard of C-F NDS; however, many participants remained unfamiliar with alternative tobacco products. The most widely recognized product was vaping devices, whereas other variants showed limited recognition.

### 3.7. Comparison of Smoking Habits According to Socio-Demographic Data and Knowledge

The comparative test results presented in Table 6 showed that smoking status did not differ significantly by age (*p* = 0.095) or education level (*p* = 0.610). However, smoking status differs significantly according to length of service (*p* = 0.000 < 0.05) and level of knowledge (*p* = 0.000 < 0.05).

Variables included in the multivariate analysis were those found to be significant based on the comparative test results presented in Table 7. Using multinomial logistic regression, a statistically significant association was observed between length of military service and smoking habits (*p* = 0.005; 95% CI: 0.282–0.800). The most significant effect was observed among participants with 1–5 years of service (*p* < 0.05). In terms of odds ratios, smoking habits increased the risk by 1.8 times among those with 11–15 years of military service.

The most significant knowledge level in influencing smoking habits was a good level of knowledge (*p* < 0.05).

## 4. Discussion

The findings of the present study demonstrated a high prevalence of current smokers among military marines (44.8%), which exceeded the civilian smoking rates of 33.8% in adults and 12.7% in adolescents, as reported in the Indonesian National Report on Basic Health Research [12]. These findings are consistent with previous studies indicating a high prevalence of tobacco use in military populations, particularly in naval services, which contributes to elevated smoking rates and increasing trends across several units [3,8,9,13]. This underscores the heightened risk within military environments.

The age distribution of current smokers in the present study was dominated by younger individuals (20–29 years). While this may partly reflect the larger number of survey participants in this age group, smokers consistently outnumbered non-smokers across all age categories. In Germany, smoking prevalence is higher among younger military personnel than among older personnel [14]. Similarly, in South Korea, the highest smoking prevalence was initially observed in the 22–24 age group [15]. Among young Greek male military recruits, smoking prevalence was also high, with most individuals initiating smoking between the ages of 10 and 20 [16].

Dolich et al. (2021) identified several critical contributing factors, including negative social environment influences (such as parental and peer smoking), psycho-emotional stress, and independent living away from parents [17]. Yeji Lee et al. (2019) further demonstrated that stress levels, secondhand smoke exposure, and social interactions were strongly correlated with smoking behaviors in young adults [18].

The education level of smokers in this study was predominantly lower. Research across multiple countries reveals complex relationships between education levels and smoking behaviors. In the U.S. military, smoking prevalence is higher among personnel with educational attainment below a bachelor’s degree [19]. A study across 21 European nations found that lower education was associated with higher daily smoking rates [20]. However, in the present study, education level was not statistically associated with smoking habits.

Previous research in military populations had consistently demonstrated significant associations between age, education, and smoking behaviors. Younger military personnel tend to exhibit higher smoking rates compared to older service members, a pattern observed across multiple studies [14,21]. In addition, educational attainment has shown a strong inverse relationship with smoking, with lower levels of education being associated with increased smoking prevalence.

According to the comparative test and multivariate analysis conducted in this study, length of military service was significantly associated with smoking habits. Although the largest number of participants had served for 1–5 years, comparative analysis showed that participants with more than 15 years of service represented a higher proportion of current smokers. Furthermore, individuals with 11–15 years of military service had 1.8 times greater odds of smoking compared to the reference group. Regarding knowledge level, a higher level of knowledge was found to significantly influence smoking habits. These findings suggest that the length of military service may significantly link to smoking behavior among personnel.

An extended duration of military service was associated with a higher propensity for sustained smoking habits. These findings suggest length of military service may be significantly associated with smoking behavior among personnel. An extended duration of military service was associated with a higher propensity for sustained smoking habits. These findings align with prior research demonstrating that military service is associated with increased smoking rates and longer smoking durations across the life course [22]. A similar association was observed among older Vietnamese military personnel, where military service significantly increased the risk of smoking initiation [23].

The association between smoking and service duration appears strongest among personnel with a prior smoking history, who demonstrate a substantially elevated risk of relapse, as well as among those exposed to combat or prolonged deployments (>9 months), enlisted personnel at lower pay grades, individuals approaching service separation, and Navy personnel relative to their Air Force counterparts [7,8,19].

Since vape/e-cigarettes are classified as tobacco products, their use among Marines was also evaluated as part of smoking behavior in this study. Most smokers primarily used conventional combustible cigarettes, while only a small proportion were e-cigarette users or dual users. This may reflect the relatively limited adoption of e-cigarettes, which are available only in certain locations and may still be perceived as less economical compared to conventional cigarettes.

Recent studies, however, have highlighted the increasing prevalence of e-cigarette use among U.S. military personnel, particularly among younger service members. While conventional cigarette use in the military declined from 24% in 2011 to 13.8% in 2015, e-cigarette use increased to 12.4% during the same period [24]. Similarly, the Health Related Behavior Survey reported an e-cigarette use prevalence of 16.2% [25]. This trend was especially pronounced among individuals aged 17–24 years, of whom 22.8% reported e-cigarette use [24]. Notably, e-cigarette use among new Air Force recruits increased substantially, reaching 15.3% in 2018 [26]. Factors associated with higher e-cigarette use included younger age, lower military rank, concurrent tobacco use, and lower perceived harm [27].

Despite the well-established health impacts of smoking, and although more than 90% of smokers in this study had attempted to quit and expressed confidence in their ability to stop smoking within six months if they chose to do so, they continued to smoke. Smoking cessation remains a significant challenge. Only about half of U.S. adult smokers attempt to quit annually, and only 7.5% achieve sustained cessation [28,29]. Similar trends have been observed in Malaysia, where 49% of smokers attempted to quit in the past year, yet only 31.4% were successful [30].

These outcomes may be attributed to numerous barriers to smoking cessation, including addiction, stress, and deeply ingrained habits [31]. The military environment may further exacerbate these challenges, as work-related stressors and the desire to conform to unit norms can contribute to increased smoking intensity and nicotine dependence [32]. In the present study, most current smokers reported no specific reason for initiating cigarette use. This may reflect an environment in which smoking is highly normalized and broadly perceived as acceptable, thereby reducing the need for conscious justification of the behavior. Nevertheless, more than one-quarter of smokers reported using cigarettes as a means to relieve stress and enhance concentration at work.

In the present study, tooth staining and halitosis were the most commonly reported oral health impacts associated with smoking. Smoking is a significant contributor to tooth staining, as toxins from tobacco smoke accumulate within the porous structure of tooth enamel [33]. Studies have shown that even light smokers (1–4 cigarettes per day) experience stain formation on their teeth [34]. Furthermore, smoking is a major risk factor for halitosis, a condition characterized by unpleasant breath odor [35]. Cigarette smoke disrupts the oral bacterial community, leading to increased production of volatile sulfur compounds in periodontal pockets [36].

Delayed wound healing and gingival changes were less frequently reported in the present study; this may be attributed to good oral hygiene practices or to the masking effect of smoking on gingival tissues. Previous research has demonstrated that smoking obscures the actual periodontal condition, as tobacco use significantly impairs vascular health and function through multiple mechanisms, resulting in reduced gingival bleeding. Consequently, this may create the false impression that the gums are healthy, when in fact the underlying tissue structures remain susceptible to disease [37].

The level of knowledge regarding smoking in this study is generally good, as most participants had received information about smoking and its associated risks, particularly its effects on the oral cavity. Participants were also aware that nicotine in cigarettes was perceived as a major contributing factor to oral health problems, ranging from tooth staining to oral cancer, although current evidence indicates that tar produced from the combustion of conventional cigarettes is primarily responsible for many smoking-related diseases [38]. Despite 71% of participants demonstrating “good” knowledge, many continued to smoke, suggesting the presence of a knowledge–behavior gap. A study among students at Riyadh Elm University sheds further attention on the significant prevalence of smoking and the knowledge gap regarding its major risks. These findings emphasize the necessity for focused and integrated tobacco control interventions that concentrate on correcting specific misconceptions, boosting self-reliance to quit and extending the provision of accessible smoking cessation assistance to encourage healthier lifestyle habits [39].

Smoking behavior among military personnel is influenced by multiple factors operating at the individual, interpersonal, and organizational levels, including educational attainment, duration of service, family history of smoking, and knowledge related to smoking [10]. Socioecological factors, including stress, work-related stressors, and smoking for unit social integration, are associated with increased smoking intensity and nicotine dependence [32].

Military-related factors, including era of service and duration of service, also influence smoking behaviors [21]. Research on the psychological aspects of smoking and cessation in military populations has demonstrated complex relationships between mental health and tobacco use. Among United Kingdom military personnel, smoking initiation has been associated with psychological distress and deployment experiences [40]. In active-duty United States military personnel, smoking intensity and nicotine dependence have been linked to smoking as a means of unit social integration, stress-related smoking, and work-related stressors [32]. In the Chinese Navy, smoking prevalence exceeds that of both civilian populations and other military branches, with stress relief and social needs identified as primary motivators [10]. Similarly, social needs and stress alleviation have been reported as the main reasons for smoking in military settings [9]. Other factors associated with increased smoking among military personnel include general life stressors [41]. Factors contributing to high smoking rates in military populations include stress alleviation, social needs, and targeted marketing by tobacco companies [10]. Although there has been an overall reduction in tobacco use within military settings, recent years have shown an increasing trend, highlighting the need for improved smoking cessation counseling and intervention strategies [3].

In the present study, the level of knowledge was found to be associated with smoking behavior. This survey also included questions regarding vaping and electronic cigarettes, as these products have the potential to reduce the adverse health effects associated with combustible cigarette use among individuals who find it difficult or are unwilling to quit smoking. More than half of the participants reported being familiar with combustion-free nicotine delivery systems (C-F NDS). Evidence suggests that the availability and marketing of C-F NDS vary according to neighborhood socioeconomic characteristics, with inexpensive combustible tobacco products being more prevalent in low-income and minority areas, whereas potentially less harmful non-combustible alternatives are more accessible in higher-income areas [42].

The initiation and continued use of C-F NDS among young adults are influenced by factors such as peer pressure, flavoring, and stress management, whereas motivations for cessation include family responsibilities and health concerns [43]. A systematic review has indicated that C-F NDS, including electronic cigarettes, smokeless tobacco, and nicotine replacement therapy, may moderately reduce daily cigarette consumption and potentially support smoking cessation attempts, with fewer adverse events compared to conventional combustible cigarettes [44].

Smoking cessation remains the most effective strategy for reducing smoking-related health risks. Health-promoting programs may contribute to improvements in smoking-related outcomes [35]. Following the implementation of a military health promotion program, a reduction in cigarette smoking prevalence among military personnel in Taiwan was observed. These findings suggest that more active interventions and structured health promotion programs are needed for smoking prevention and cessation in military populations. In addition, military institutions should develop specific, targeted strategies to address tobacco use effectively [13]. Overall, these findings highlight the importance of comprehensive and targeted smoking prevention and cessation programs for military personnel.

However, this study has limitations concerning the sampling that enables a biased response, regarding the self-reported data, and the representativeness of the subjects, as it depends on the availability and accessibility of the survey. Their work assignments cannot be precisely predicted in terms of location and timing; at times, they are deployed to areas that are very remote or even lack internet access, preventing them from participating in this survey. In addition, this survey was conducted exclusively among the Marine military population.

## 5. Conclusions

This study demonstrated a high prevalence of tobacco use among military marines, particularly within the Marine Corps, despite generally high levels of knowledge regarding tobacco smoking. A knowledge gap was still evident in relation to smoking behavior. The most frequently reported oral health impacts among smokers were tooth staining, halitosis, and taste impairment. Duration of military service and level of knowledge were significantly associated with smoking behavior.

## Figures and Tables

**Table 1:** General characteristic and smoking status (*n* = 475).

Variable	F (%)
**Characteristics**	
**Age (years)**	
<20	20 (4.2)
20–29	225 (47.4)
30–39	121 (25.5)
40–49	99 (20.8)
50–59	10 (2.1)
** ** **Sex**	
Male	475 (100)
Female	0 (0)
** ** **Military service (years)**	
1–5	178 (37.5)
6–10	87 (18.3)
11–15	33 (6.9)
>15	177 (37.3)
** ** **Education level**	
Senior high school or equivalent	448 (94.5)
College	25 (5.2)
Postgraduate and above	2 (0.4)
** ** **Smoking status**	
Current smokers	213 (44.8)
Former smokers	122 (25.7)
Never smokers	140 (29.5)

**Table 2:** Smoking behavior.

Smoking Behavior	F (%)
**Current smokers**	
** ** **Type of cigarette**	
Conventional Cigarette	188 (88.3)
Electric Cigarette	3 (1.4)
Conventional & E-cigarette	22 (10.3)
NA	-
** ** **Duration of smoking (years)**	
<1	15 (7.0)
1–2	18 (8.5)
3–5	41 (19.2)
5–10	61 (28.6)
>10	75 (35.2)
NA	3 (1.4)
** ** **Smoking frequency/day**	
<5	62 (29.1)
5–10	79 (37.1)
>10	69 (32.4)
NA	3 (1.4)
** ** **Total smoking time/day (hours)**	
<1	55 (25.8)
1–2	69 (32.4)
3–5	60 (28.2)
>5	27 (12.7)
NA	2 (0.9)
** ** **Number of cigarettes smoked/day**	
<5	52 (24.4)
5–10	82 (38.5)
>10	75 (35.2)
NA	4 (1.9)
** ** **Purpose of smoking**	
Promote concentration on work	46 (21.6)
Relieve stress	60 (28.2)
Enjoy the smell (of e-cig liquid) without harming your health	2 (0.9)
Have no reason	105 (49.3)
**Former smokers**	
** ** **Type of cigarette**	
Conventional Cigarette	99 (81.1)
Electric Cigarette	3 (2.5)
Conventional & E-cigarette	19 (15.6)
NA	1 (0.8)
** ** **Duration of smoking (years)**	
<1	43 (35.2)
1–2	21 (17.2)
3–5	22 (18.0)
5–10	12 (9.8
>10	22 (18.0)
NA	2 (1.6)
** ** **Total smoking time/day (hours)**	
<1	30 (24.6)
1–2	30 (24.6)
3–5	28 (23.0)
>5	15 (12.3)
NA	19 (15.6)

**Abbreviations**: NA, not available.

**Table 3:** Cessation attempts and history of current smokers.

Cessation Attempts and History	F (%)
**Had attempted to quit**	
Yes	191 (89.7)
No	19 (8.9)
NA	3 (1.4)
**Had received advice to quit smoking**	
Yes	124 (58.2)
No	86 (40.4)
NA	3 (1.4)
**Believe they will be able to quit in six months if they decide to quit smoking**	
Yes	159 (74.6)
No	50 (23.5)
NA	4 (1.9)
**Had experienced negative health impacts due to smoking**	
Yes	125 (58.7)
No	85 (39.9)
NA	3 (1.4)
**Health expectancy related to smoking cessation**	
Yes	194 (91.1)
No	14 (6.6)
NA	6 (2.3)

**Table 4:** Smoking impact on oral health among current and former smokers.

Smoking Impact on Oral Health	F (%)
**Halitosis**	
Yes	272 (81.2)
No	54 (16.1)
NA	9 (2.7)
**Tooth Staining**	
Yes	285 (85.1)
No	40 (11.9)
NA	10 (3.0)
**Impaired Taste**	
Yes	169 (50.4)
No	156 (46.6)
NA	10 (3.0)
**Delay wound healing**	
Yes	124 (37.0)
No	201 (60.0)
NA	10 (3.0)
**Gingival soreness**	
Yes	126 (37.6)
No	200 (59.7)
NA	9 (2.7)
**Gingival bleeding**	
Yes	114 (34.0)
No	211 (63.0)
NA	10 (3.0)
**Gingival swelling**	
Yes	112 (33.4)
No	213 (63.6)
NA	10 (3.0)

**Table 5:** Knowledge regarding smoking.

Knowledge Regarding Smoking	F (%)	
Good (12–16)	339 (71.4)	
Fair (6–11)	100 (21.1)	
Poor (0–5)	36 (7.6)	
**Statement on smoking**	**Yes—**	**No—**
	***N*** **(%)**	***N*** **(%)**
I had received information about smoking and its risks	462 (97.3)	13 (2.7)
Smoking may cause changes in the oral cavity (hard tissue or soft tissue)	395 (83.2)	80 (16.8)
The number of cigarettes affects the onset of changes in the oral cavity	374 (78.7)	101 (21.3)
Smoking is not cool	408 (85.9)	67 (14.1)
Nicotine in cigarettes is the chemical that most contributes to oral cancer	400 (84.2)	75 (15.8)
Cigarette smoking may lead to apoplexy	254 (53.5)	221 (46.5)
Cigarette smoking may lead to lung cancer	410 (86.3)	65 (13.7)
Cigarette smoking may lead to stained teeth	438 (92.2)	37 (7.8)
Cigarette smoking may lead to premature aging	329 (69.3)	146 (30.7)
Cigarette smoking may lead to coronary heart disease	390 (82.1)	85 (17.9)
Cigarette smoking may lead to mouth cancer	386 (81.3)	89 (18.7)
Cigarette smoking may lead to impotence in male smokers	370 (77.9)	105 (22.1)
Passive smoking may lead to lung cancer in non-smokers	398 (83.8)	77 (16.2)
**Statement about C-F NDS**		
Heard about C-F NDS	297 (62.5)	178 (37.5)
Recognize about C-F NDS	261 (54.9)	214 (45.1)
**Variants of C-F NDS that are known:**		
Vape	125 (26.3)	
Heated tobacco product	45 (9.5)	
Nicotine patch	31 (6.5)	
Nicotine gum	35 (7.4)	
Snus	5 (1.0)	
Do not familiar	234 (49.3)	
**Total *N* (%)**	**213 (44.8)**	

**Table 6:** Comparison of Smoking Status According to Socio-demographic Data and Knowledge.

Characteristics	Current Smokers	Former Smokers	Never Smokers	*p*-Value
**Age**				
<20	6 (2.8)	7 (5.7)	7 (5.0)	0.095
20–29	86 (40.4)	61 (50.0)	78 (55.7)	
30–39	65 (30.5)	28 (23.0)	28 (20.0)	
40–49	50 (23.5)	23 (18.9)	26 (18.6)	
50–59	6 (2.8)	3 (2.5)	1 (0.7)	
**Education Level**				
Senior high school or equivalent	201 (94.4)	115 (94.3)	132 (94.3)	0.610
College	10 (4.7)	7 (5.7)	8 (5.7)	
Postgraduate and above	2 (0.9)	0 (0)	0 (0)	
**Military service (years)**				
1–5	58 (27.2)	57 (46.7)	63 (45.0)	0.00
6–10	42 (19.7)	16 (13.1)	29 (20.7)	
11–15	22 (10.3)	5 (4.1)	6 (4.3)	
>15	91 (42.7)	44 (36.1)	42 (30.0)	
**Knowledge**				
Good	129 (60.6)	95 (77.9)	115 (82.1)	0.00
Fair	58 (27.2)	21 (17.2)	21 (15.0)	
Poor	26 (12.2)	6 (4.9)	4 (2.9)	

**Table 7:** Multivariate Analysis on Current Smokers.

Variable	Beta Coefficient	SE	*p*-Value	OR	95% Lower	CI OR Upper
**Military service (years)**						
1–5	−0.745	0.266	0.005	0.475	0.282	0.8
6–10	−0.307	0.313	0.327	0.736	0.399	1.358
11–15	0.624	0.506	0.218	1.866	0.692	5.035
>15	-	-	-	-	-	-
**Knowledge**						
Good	−1.65	0.557	0.003	0.192	0.064	0.572
Fair	−0.774	0.6	0.197	0.461	0.142	1.494
Poor	-	-	-	-	-	-
**Intercept**	2.065	2.065	0.559	0.00	-	-

## Data Availability

The original contributions presented in this study are included in the article. Further inquiries can be directed to the corresponding author.
